# Abnormal methylation caused by folic acid deficiency in neural tube defects

**DOI:** 10.1515/biol-2022-0504

**Published:** 2022-12-22

**Authors:** Rui Cao, Jun Xie, Li Zhang

**Affiliations:** Department of Biochemistry and Molecular Biology, Shanxi Key Laboratory of Birth Defect and Cell Regeneration, Key Laboratory for Cellular Physiology of Ministry of Education, Shanxi Medical University, No. 56, Xinjian South Road, Yingze District, Taiyuan, Shanxi Province, China; Shanxi Key Laboratory of Pharmaceutical Biotechnology, Shanxi Biological Research Institute Co., Ltd, Taiyuan, China; Department of Hepatobiliary and Pancreatic Surgery and Liver Transplant Center, The First Hospital of Shanxi Medical University, No. 56, Xinjian South Road, Yingze District, Taiyuan, Shanxi Province, China

**Keywords:** neural tube defects, DNA methylation, histone methylation, m6A RNA methylation, folic acid

## Abstract

Neural tube closure disorders, including anencephaly, spina bifida, and encephalocele, cause neural tube defects (NTDs). This congenital disability remained not only a major contributor to the prevalence of stillbirths and neonatal deaths but also a significant cause of lifelong physical disability in surviving infants. NTDs are complex diseases caused by multiple etiologies, levels, and mechanisms. Currently, the pathogenesis of NTDs is considered to be associated with both genetic and environmental factors. Here, we aimed to review the research progress on the etiology and mechanism of NTDs induced by methylation modification caused by folic acid deficiency. Folic acid supplementation in the diet is reported to be beneficial in preventing NTDs. Methylation modification is one of the most important epigenetic modifications crucial for brain neurodevelopment. Disturbances in folic acid metabolism and decreased *S*-adenosylmethionine levels lead to reduced methyl donors and methylation modification disorders. In this review, we summarized the relationship between NTDs, folic acid metabolism, and related methylation of DNA, imprinted genes, cytoskeletal protein, histone, RNA, and non-coding RNA, so as to clarify the role of folic acid and methylation in NTDs and to better understand the various pathogenesis mechanisms of NTDs and the effective prevention.

## 1 Introduction

Abnormal development of the central nervous system (CNS) caused by neural tube defects (NTDs), such as anencephaly, spina bifida, and encephalocele, is a major contributor to stillbirths and neonatal deaths. It is also a significant cause of lifelong physical disability in surviving infants. NTDs are common clinical birth defects caused by incomplete or disordered neural tube closure in embryos, with an incidence of 1.86‰ in humans [1]. Human NTDs are associated with genetic and environmental actors, such as folic acid deficiency, which is an important cause of NTDs [2]. Epigenetics is a branch of genomics that refers to the heritability of gene expression without modifying the DNA sequence [3]. DNA methylation is a common epigenetic modification involved in neural tube development, but its underlying mechanism remains unclear [4]. This article reviews the research progress on the etiology and mechanism of NTDs induced by methylation modification caused by folic acid deficiency.

## 2 NTDs

The neural tube is the embryonic precursor of the CNS, which eventually develops into the brain, spinal cord, neurohypophysis, and pineal gland. In the early stages of embryonic development, neuroectoderm cells proliferate, invaginate, and eventually migrate from the surface of the ectoderm to form the primary neural tube [5]. Invaginated cells sag to form cell cords, generating a secondary neural tube. Mouse neural tube closure is initiated at E8.5, including three sites: the first closure site is at the junction of the hindbrain and cervical, the second is located at the junction of the forebrain and midbrain, and the third is located at the rostral side of the forebrain. The entire process is carried out along the spinal cord and completed at E10.5 [6]. In humans, neural tubes close between 21 and 28 days after conception [7].

NTDs are one of the leading causes of abortion, infant death, and children with lifelong disabilities [8]. Worldwide, the incidence of NTDs is 1.86‰, and in northern China, it reaches 13.9% [9]. In addition, some studies have found that 1 in 10 babies with NTDs died in their first year of life [10]. The etiology of NTDs involves genetic and environmental factors, of which genetic factors account for approximately 20%, and non-genetic factors account for approximately 80% of all cases with NTDs [11]. Indeed, more than 240 genes have been identified which are involved in neural tube closure [12], and this number is expected to increase.

Folic acid deficiency in pregnant women increases the risk of developing NTDs. Among all the folic acid-related genes, 5,10-methylenetetrahydrofolate reductase (*MTHFR*) is a focal point in the research of the NTDs field. *MTHFR* C677T and A1298C gene variants contribute to the increased risk for NTDs, but this association only appears in specific populations [13]. The combination of the three wild-type alleles *MTHFR* (C677T, A1298C) and methionine synthase reductase (*MTRR*) A66G has increased four-fold the incidence of NTDs [14]. In summary, NTDs are multi-gene, multi-level, and multi-mechanism diseases.

## 3 Methylation and NTDs

Methylation refers to the addition of a methyl group from an active methyl donor to a compound. In this process, various methyl compounds can be formed directly or by chemical modification of proteins or nucleic acids. Methylation modification mainly includes the methylation of DNA, histones, RNA, and imprinted genes [3,15]. It is a crucial research branch of epigenetics research and plays an indispensable role in embryonic development by regulating cell division, proliferation, gene expression, homocysteine balance, and genome stability and integrity [16,17]. At present, methylation is believed to be closely related to cancer, aging, senile dementia, defects in neural tube development, and many other diseases [18,19,20,21,22,23].

**Figure 1:**
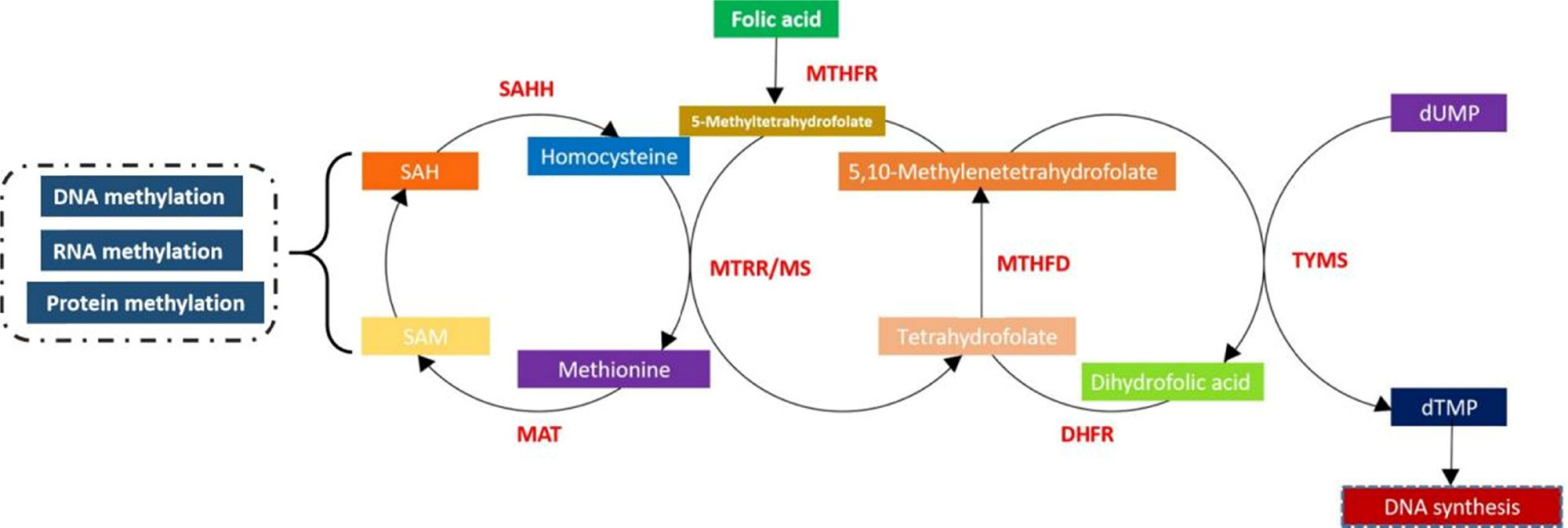
The folic acid cycle and the relationship with methylation and DNA synthesis. MTHFR, 5,10-methylenetetrahydrofolate reductase; MTRR, methionine synthase reductase; MS, methionine synthetase; MAT, methionine adenosyltransferase; SAHH, *S*-adenosyl-l-homocysteine hydrolase; MTHFD, methylenetetrahydrofolate dehydrogenase; DHFR, dihydrofolate reductase; TYMS, thymidylate synthetase; SAM, *S*-adenosylmethionine; and SAH, *S*-adenosyl-l-homocysteine.

Tetrahydrofolate (THF) is a coenzyme of one-carbon unit metabolism, synthesized by folic acid [24]. This coenzyme is mainly involved in de novo nucleotide synthesis, homocysteine remethylation, and intracellular methylation reactions. Folic acid deficiency leads to a decrease in THF and other important coenzymes, resulting in a series of abnormalities such as the metabolism disorder of one carbon unit and single nucleotide and GTP reduction, which inhibit the redox chain reactions [25]. Methylation then changes, which causes a decrease in methyl compounds and has an adverse impact on protein translation. This process induces the dysfunction of tissues and organs, leading to the occurrence of diseases [26]. The nitrogen 5-trimethyl-tetrahydrofolate (N_5_–CH_3_–FH_4_) in the one-carbon unit provides a methyl group for the homocysteine to generate methionine. The methionine is then activated to *S*-adenosylmethionine (SAM), the methyl donor in mammals. Therefore, the lack of folic acid *in vivo* also affects the metabolism of N_5_–CH_3_–FH_4_, resulting in decreased SAM levels and an insufficient supply of methyl donors, hindering the methylation of DNA, RNA, and proteins [17]. Figure 1 shows the folic acid cycle and the relationship with methylation and DNA synthesis.

In summary, many studies have shown that epigenetic mechanisms may play an essential role in neural tube development, and abnormal methylation may be the principal reason for NTDs [27].

## 4 DNA methylation and NTDs

DNA methylation is a major epigenetic modification of the genome and regulates gene expression [3]. DNA methylation refers to the covalent bonding of a methyl group at the cytosine-5-carbon site of CpG dinucleotide under the action of a DNA methylation transferase. DNA methyltransferase (Dnmt) mainly consists of three forms, Dnmt1, Dnmt2, and Dnmt3, which catalyze DNA methylation [28].

Genome-wide reprogramming of DNA methylation patterns occurs during early embryonic development [29]. Before the first mitosis of the mammalian zygote, genomic DNA from parents is demethylated and remethylated after embryo implantation [30]. Subsequently, demethylation and transcription of susceptibility genes occur. In this process, incorrect methylation modifications can induce NTDs [31].

During embryonic development, Dnmt1, Dnmt3a, and Dnmt3b are highly expressed, among which Dnmt1 maintains genomic DNA methylation, while Dnmt3a and Dnmt3b are responsible for establishing the genomic DNA methylation state [32]. It was found that the DNA methyltransferases Dnmt1, Dnmt3a, and Dnmt3b regulate 5-methylcytosine (5-mC), and the methylation level of the 5-mC in NTDs embryonic liver tissue was decreased compared to normal embryonic tissue [33,34,35]. Knockdown of Dnmt3b in mice can alter DNA methylation and cause various developmental defects [36]. In addition, excessive oxidative stress increases the Dnmt3b activity, resulting in altered methylation of the paired box 3 (*Pax3*) gene, which has been shown to be involved in the occurrence of NTDs [37].

Changes in DNA methylation levels were found in various NTD mouse models. The methionine cycle inhibitors ethionine and cycloleucine interfere with mouse embryos *in vitro*, leading to a significantly decreased conversion of SAM to *S*-adenosylhomocysteine, inhibition of methylation, and significantly increased incidence of NTDs [26]. The NTDs animal model was successfully established after the inhibitor ethionine intervention in E7.5d pregnant mice. It was also found that the DNA methylation level of embryonic tissue in the intervention group was significantly decreased [38]. Lower DNA methylation levels were also found in methotrexate-induced NTD embryos [39]. Similarly, the important role of methylation in the occurrence of NTDs has also been confirmed in chick embryo animal models [40].

Other studies have shown that gene methylation, such as the methylation of vault RNA 2-1 (*VTRNA2-1*), hypomethylation of *Caspase-8* (*CASP8*), and demethylation of nucleosome assembly protein 1-like 2 (*NAP1L2*), is associated with NTDs [41,42,43]. A previous study used methylation-specific multiplex ligation-dependent probe amplification to detect O6-methylpurine-DNA methyltransferase (*MGMT*) and show that its methylation is closely related to NTDs [44]. Overall, DNA methylation plays a crucial role in neural tube development in mammals.

The aforementioned studies suggest that DNA methylation inhibition can cause NTDs, and appropriate methylation levels play a crucial role in neural tube development in mammals [45]. A previous study has shown that folic acid levels are directly related to genomic DNA methylation, and its supplementation can reverse DNA hypomethylation to varying degrees [46]. Supplementing and increasing the availability of folic acid can prevent NTDs by providing methyl donors to promote DNA methylation. However, the mechanisms of DNA methylation at cellular, molecular, or genetic levels during neural tube development remain unclear. Large-scale high-throughput techniques and knockdown experiments are needed to confirm epigenetically regulated genes and signal pathways that are crucial for NTDs, thereby providing a scientific basis for targeted intervention and prevention of NTDs.

Differentially methylated CpG sites were found in anencephaly cases to controls based on methylation450 (450k) array. The mechanisms and the pathways of the sites cg24666096, cg10988628, and cg02413938, which were involved in PARP1, ESPNL, and other genes, are still unclear [47]. In addition, the effect of folic acid on the methylation profiles is an important aspect in the fields of NTDs research. In a recent study, a total of 1939 differentially methylated genes (DMG) were detected in the folate-deficient diet group and 1498 DMG in the folate-supplemented diet group compared with the folate-normal diet group. Among them, the genes and pathways related to neural development were as follows: *Wnt10a, Isl1, Neurog1, Onecut1* in signaling pathways regulating pluripotency of stem cells; *Irs2, Irs3, Nrbf2, Pten, Rb1cc1, Rragd, Ulk2* in autophagy pathway; *Nedd4l, Cacna1d, Cldn17, Ezr, Itgb1, Slc9a3r1* in tight junction pathway; *Grin2d, Adcy3, Adcy7, Cacna1d, Cacna1h, Gnal, Phkg2* in the calcium signaling pathway, and so on. These can be candidate genes and pathways for future studies [48].

## 5 Imprinted gene methylation and NTDs

Imprinted genes are a special class of gene clusters regulated by epigenetic modifications [49]. Currently, there are 200 imprinted genes confirmed or predicted in humans, and approximately 140 are shown to be present in mice [50]. Imprint formation in mammalian development mainly includes three processes: demethylation, remethylation of imprints, and methylation maintenance [51]. Imprinted genes are divided into maternal and paternal imprinted genes. Maternally imprinted genes mainly promote embryo development, such as the insulin-like growth factor 2 (*IGF2*). Loss of maternal imprinting can lead to intrauterine growth and developmental retardation in fetuses. The primary function of paternal imprinting genes is to inhibit embryonic development. For example, the imprinting deletion of the long non-coding RNA H19 (H19) leads to excessive fetal growth [52]. Researchers found that in fetuses with NTDs, methylation levels of H19DMR1 and IGF2 differentially methylated regions DMR0 (IGF2DMR0) were significantly higher than those in normal fetuses [53,54].

Many studies in folate-deficient NTDs human samples and NTDs animal models have shown that folate deficiency is closely related to the regulation of imprinting. A prospective cohort study in the United Kingdom found that the presence of folic acid supplements after 12 weeks of gestation elevated *IGF2* methylation levels and decreased paternally expressed gene 3 (*Peg3*) methylation levels. The results showed that folic acid treatment after 12 weeks of gestation could affect the methylation of imprinted genes in offspring [55]. Other researchers have found altered methylation levels of imprinted genes in folate-deficient NTDs mouse embryos [33,35,56]. Aberrant DNA methylation in GNAS imprinting cluster was found in clinical NTDs samples with low folate concentrations [57].

In summary, studies have shown that imprinted genes play an essential role in regulating the growth and development of embryos and fetuses after birth and further affect body behavior and brain function [51,58]. But the specific pathogenesis is not fully understood, and further studies are needed. We could construct NTDs mouse models with different folic acid metabolism disorders: folic acid-THF, nucleotide triphosphate (NTP)-deoxynucleoside triphosphate (dNTP), deoxyuridine monophosphate (dUMP)-deoxythymidine monophosphate (dTMP) and SAM-SAH, to detect CNV changes of imprinted genes and its potential relationship with NTDs.

## 6 Cytoskeletal protein methylation modification and NTDs

Among the cytoskeletal components, actin, tubulin, and neurofilament L are methylated during embryo development [59]. SAM provides methyl groups during neural tube closure to generate active sites for actin and myosin binding, and these sites have a highly conserved 3-methylhistidine residue [60]. In the primary neural tube formation process, signals for neural tube development are sensed by the cytoskeleton and transmitted to adjacent cells [61]. Folic acid deficiency leads to reduced methylation of key sites in cytoskeletal elements, failure of localization of cytoskeletal elements in neural tissues, and failure of cell contraction and movement, which affect cell invaginating during development and further results in NTDs [60].

## 7 Histone methylation modification and NTDs

Histone modification is another area of epigenomic research centered on histone methylation. There are 24 known histone methylation sites, including 17 lysine residues and 7 arginine residues [62]. Histone methylation modifications can regulate gene expression, thereby affecting embryonic development [63]. Some studies have reported that abnormal histone H3 lysine 72 trimethylation (H3K27me3) levels may be a risk factor for NTDs. Reduced H3K27me3 can leads to abnormal Hox gene expression in NTD [64], and increased H3K27me3 expression also might cause a disorder of folate metabolic pathway [65]. Changes in H3K79 methylation levels cause abnormal gene expression during neural development, leading to the occurrence of NTDs [66].

Reportedly, folic acid deficiency may directly affect the methylation of histones, regulating the expression of key genes and causing NTDs [67]. It was found that the modification levels of histone H3 lysine 9 trimethylation (H3K9me3) and histone H4 lysine 20 trimethylation (H4K20me3) were significantly reduced when rats were fed with a methyl-deficient diet [68]. Folic acid can regulate the expression of demethylase Jumonji-D3 (*JMJD3*/*KDM6B*) through microRNA (miRNA) and affect H3K27me3 levels [69]. Folic acid deficiency induces hypermethylation and leads to low expression of the Brachyury gene (T gene), which is involved in NTDs [70].

Knockout or mutation of some histone methyltransferase genes can also cause NTDs. In a histone-lysine *n*-methyltransferase (*Ezh2*) knockdown mouse model, germ formation was inhibited [71]. *Ezh2* knockdown chicken model showed that the neuroepithelium structure was destroyed, and the proliferation of the nerve progenitor cells was reduced [72]. Severe defects in neural tube formation, somatogenesis, and cardiac development were found in SET domain-containing 5 (*Setd5*) knockdown mouse models, as well as abnormalities in the embryonic yolk sac and placental angiogenesis [73]. The neural tube, yolk sac, and heart showed defects in H3K27 demethylase (*UTX*) homozygous mutant embryos [74].

### 7.1 N6-methyladenosine RNA methylation modification and neural tube development

**Figure 2:**
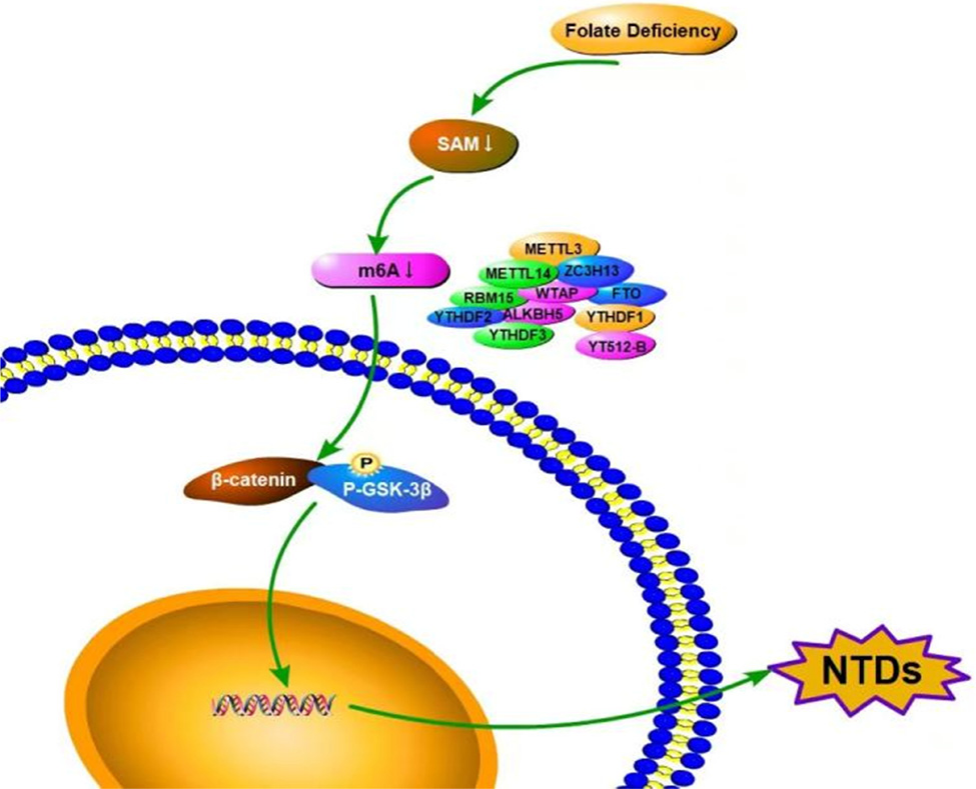
Relationship among folate deficiency, SAM, m6A modification, and NTDs.

N6-methyladenosine (m6A) RNA methylation is a new epigenetic modification similar to DNA or histone modification, which is involved in many biological processes, such as RNA splicing, protein translation, and stem cell regeneration. m6A modification is regulated by several proteins including METTL3, METTL4, ZC3H13, WTAP, RRB15, VIRMA, FTO, ALKBH5, HNRNPC, YTHDF1, YTHDF2, YTHDF3, YTHDC1, and YTHDC2 [75,76,77,78]. Increasing evidence indicates that m6A modification plays an important role in mammals. METTL3 mutations are lethal to both mammalian and plant embryos [79]. Some studies have used Si-MettL3 to interfere with oocytes. The results showed that the reduction of METTL3 levels could inhibit the mRNA translation efficiency and mitigate mRNA degradation, suggesting that reversible m6A modification plays an important role in mammalian oocyte maturation and the development of preimplantation embryos [80]. Our previous research found that m6A modification is closely related to NTDs and that METTL3 defect leads to reduced proliferation in HT-22 cells and results in excessive cell apoptosis via suppressing Wnt/β-catenin signaling pathway [81] as shown in Figure 2. Knockdown of *YTHDF2* can lead to embryo death during late embryonic development, which is mainly manifested as impaired neural development, disrupted proliferation of neural stem/progenitor cells, and disrupted neuronal differentiation [82]. The maternal genotype of demethylase FTO is associated with NTDs, which is the first identified m6A eraser [83]. In addition, abnormal m6A RNA modification can lead to developmental retardation in parthenogenetic embryos [84].

Studies have shown that in an acute lead exposure mouse model, the expression level of FTO increases, and the methylation level of m6A decreases. After the folic acid intervention, m6A methylation levels increased. Therefore, it can be inferred that folic acid is not only involved in the process of DNA methylation, but also the process of RNA methylation [85]. However, in NTDs models or samples, the mechanism of folate deficiency leading to the change of rnam6a methylation level is not clear. NTDs mouse models of SAM deficiency need to be established. MERIP sequencing method could detect the m6A regions on the whole genome level and different expressions of m6A methylase and demethylase could be analyzed. These results would provide the basis for revealing the role of methylation metabolism in the onset of NTDs.

### 7.2 Non-coding RNA methylation modification and NTDs

For non-coding RNAs, there is a significant correlation between epigenetic function and transcriptional regulation [86]. It was hypothesized that tRNA methylation could reduce ribonuclease degradation and prolong the half-life and functionality of each molecule [87,88]. At present, how the methylation of miRNA, small interfering RNA, PIWI-interacting RNAs, and RNA interacting with Piwi affects epigenetic modification has become a hotspot in the field of biological research. A previous study has shown that Mir-129-2 inhibits autophagy by directly targeting peroxisome proliferator-activated receptor γ coactivator-1α in a hyperglycemia-induced NTD model and may also induce changes in methylation modifications [89]. Altered miRNA expression level leads to abnormal neural tube development by regulating key planar cell polarity pathways [90]. Abnormal miRNA regulation exists in retinoic acid-induced NTD mouse embryos [91]. Folic acid can downregulate Mir-138 and Mir-let-7 levels through the folate receptor and regulates the H3K27ac expression level of the octamer-binding transcription factor 4 (*Oct4*) [92]. However, how the methylation modification of miRNA regulates gene expression and protein translation in folic acid-deficient animal models of NTDs requires further study. We could induce pluripotent stem cells to differentiate into neurons, glial cells, etc., to explore whether miRNA methylation regulates genes’ expression which is associated with NTDs at the cellular level. At the same time, small molecule drugs with potential clinical application value could be screened by constructing neural tube organoids.

## 8 Conclusions

**Figure 3:**
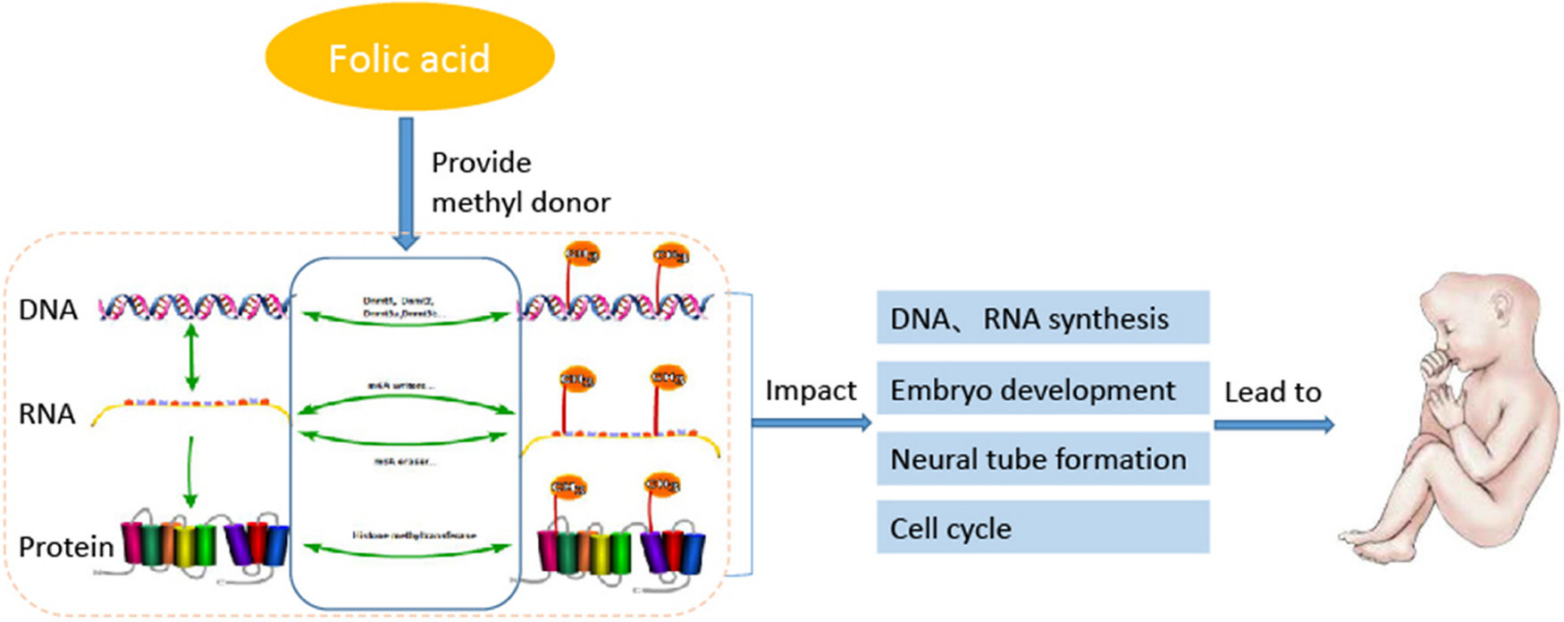
Role of folic acid in methylation and NTDs.

NTDs are severe and common congenital malformations. Folic acid plays a vital role in the development of NTDs (Figure 3). Neural tube closure is controlled by the accumulation of spontaneous and region-specific behavioral changes in many cells. Its complexity mainly depends on the discontinuity of the closure process, which is very important for explaining the diversity phenotype of human NTDs. Inhibition of the methionine cycle and methylation modification caused by changes in SAM levels also play an essential role in the occurrence of NTDs. However, how methylation modification regulates related genes and pathways leading to NTDs remains a research hotspot of great scientific value. It is necessary to conduct a comprehensive and systematic study on the biological processes of neural tube closure from the perspective of heredity, epigenetics, and environmental factors. In-depth studies of methylation modification of DNA, histones, and m6A and the related mechanisms will provide critical information for improving prevention strategies and treatment of NTDs in the future.
